# Supplementary figures and images for: Antibiotic that inhibits trans-translation blocks binding of EF-Tu to tmRNA but not to tRNA
Source: mBio. 2023 Sep 8;14(5):e01461-23. doi: 10.1128/mbio.01461-23 (PMC10653918; doi:10.1128/mbio.01461-23)

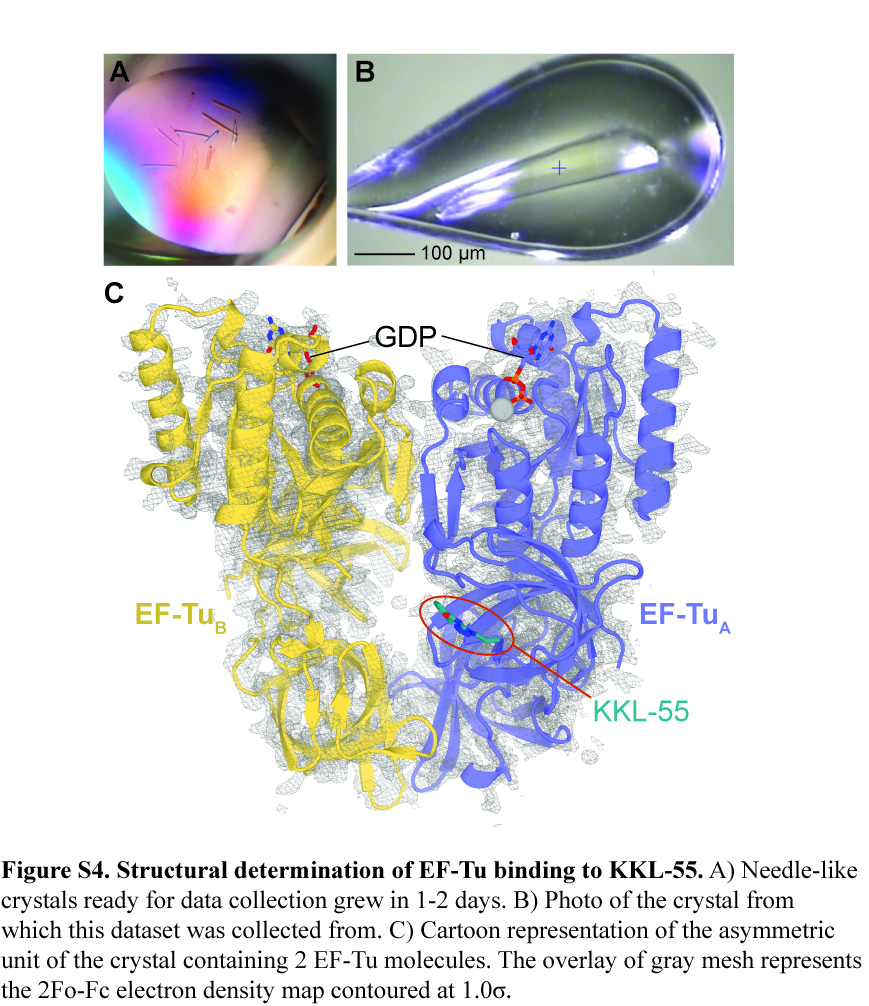

Supplement: Fig. S4 — Structural determination of EF-Tu binding to KKL-55. [file mbio.01461-23-s0004.tif]

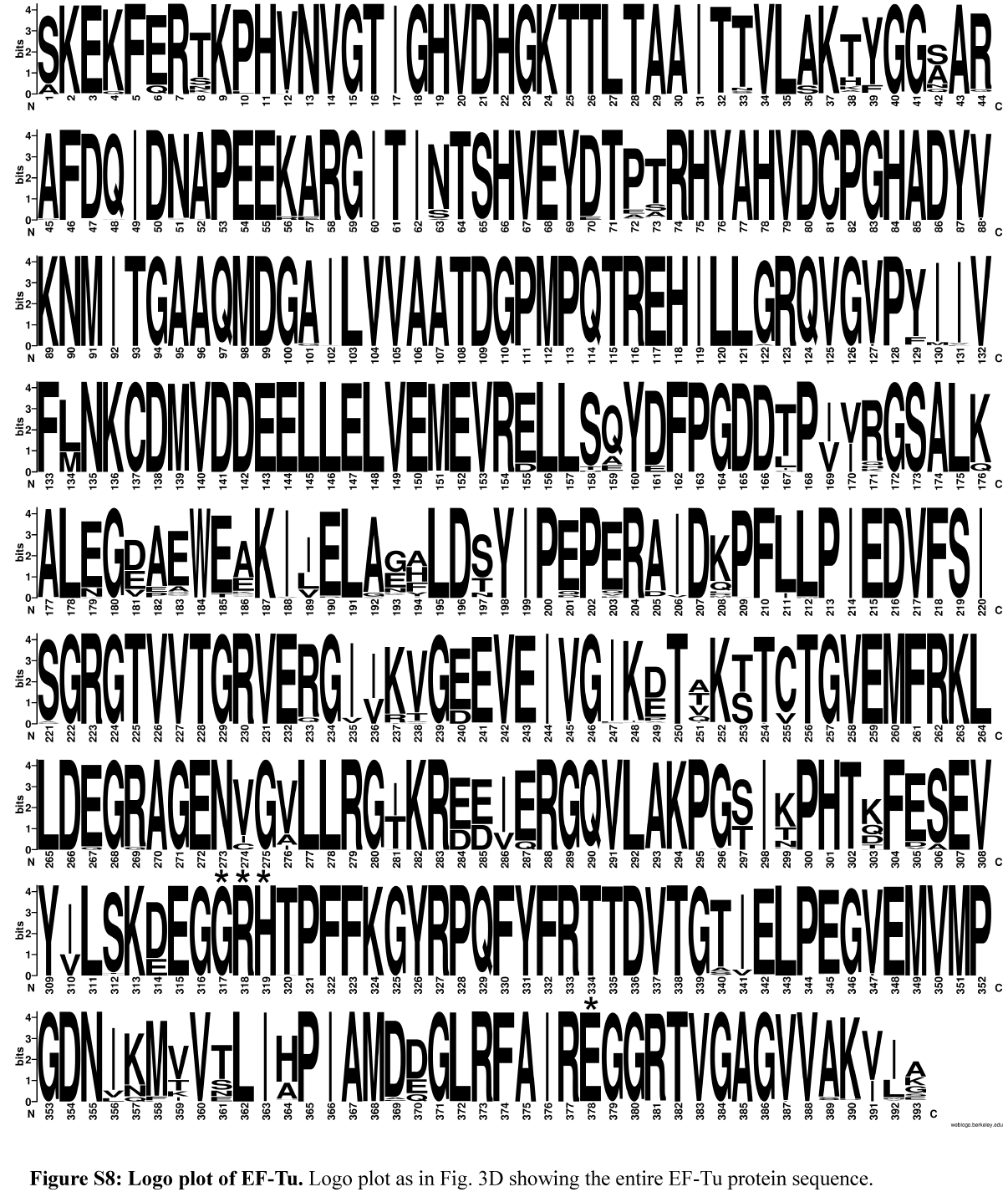

Supplement: Fig. S8 — Logo plot of EF-Tu. [file mbio.01461-23-s0008.tif]
