# Supplementary material for: Antibiotic that inhibits trans-translation blocks binding of EF-Tu to tmRNA but not to tRNA
Source: mBio. 2023 Sep 8;14(5):e01461-23. doi: 10.1128/mbio.01461-23 (PMC10653918; doi:10.1128/mbio.01461-23)
Supplement: Supplemental tables — Tables S1 to S3. [file mbio.01461-23-s0010.docx]

Table S1: Strains used in this study

| Strain | Description | Source or Reference |
| --- | --- | --- |
| *B. anthracis Sterne* | pXO1^+^, pXO2^-^ | Gift from Turnbough Lab |
| *E. coli DH5α* | cloning strain | New England Biolabs |
| JW5503 | *tolC::Kan* | (1) |
| *E. coli ∆tolC∆tufB* | Markerless with *tolC* and *tufB* deletion | This study |
| KCK571 | *E. coli* *∆tolC∆tufB ∆tufA::Kan* with WT *tufA* on pCA24N | This study |
| KCK572 | *E. coli* *∆tolC∆tufB ∆tufA::Kan* with pCA24NR318K | This study |
| KCK573 | *E. coli ∆tolC∆tufB ∆tufA::Kan* with pCA24NH319A | This study |
| KCK574 | *E. coli ∆tolC∆tufB ∆tufA::Kan* with pCA24NE378A | This study |
| JW3301 | *tufA::Kan* | (1) |
| KCK559 | DH5α with pCA24NR318A | This study |
| KCK560 | DH5α with pCA24NR318K | This study |
| KCK561 | DH5α with pCA24NR318N | This study |
| KCK562 | DH5α with pCA24NH319A | This study |
| KCK563 | DH5α with pCA24NE378A | This study |
| KCK564 | DH5α with pCA24NR318AH319AE378A | This study |
| KCK565 | *E. coli ∆tolC*∆*tufB* with WT *tufA* on pCA24N | This study |
| KCK566 | *E. coli ∆tolC∆tufB* with pCA24NR318A | This study |
| KCK567 | *E. coli ∆tolC∆tufB* with pCA24NR318K | This study |
| KCK568 | *E. coli ∆tolC∆tufB* with pCA24NR318N | This study |
| KCK569 | *E. coli ∆tolC∆tufB* with pCA24NH319A | This study |
| KCK570 | *E. coli ∆tolC∆tufB* with pCA24NE378A | This study |
| JW3301 | tufA-6XHis | (2) |
| KCK575 | Tetracycline resistance marker linked to *tufA::Kan* in genome | This study |

Table S2: Plasmids used in this study

| Plasmid | Description |
| --- | --- |
| pDHFR | Expresses DHFR off a T7 promoter; Amp^R^ |
| pCA24N-His6-tufA | IPTG-inducible expression of EF-Tu; Chlor^R^ |
| pCA24N-His6R318A | pCA24N vector expressing R318AEF-Tu-6XHis, IPTG-inducible, Chlor^R^ |
| pCA24N-His6R318K | pCA24N vector expressing R318KEF-Tu-6XHis, IPTG-inducible, Chlor^R^ |
| pCA24NHis6R318N | pCA24N vector expressing R318NEF-Tu-6XHis, IPTG-inducible, Chlor^R^ |
| pCA24NHis6H319A | pCA24N vector expressing H319AEF-Tu-6XHis, IPTG-inducible, Chlor^R^ |
| pCA24NHis6E378A | pCA24N vector expressing E378AEF-Tu-6XHis, IPTG-inducible, Chlor^R^ |
| pCA24NHis6R318AH319AE378A | pCA24N vector expressing R318AH319AE378AEF-Tu-6XHis, IPTG-inducible, Chlor^R^ |

Table S3: Oligonucleotides used in this study

| Use | Oligonucleotide | Sequence |
| --- | --- | --- |
| Fragment 1 for all mutants | tufAall_frag1_fwd | ACCATCACCATCACCCATACGTCTAAAGAAAAATTTGAACGTACAAAACCGCACGTTAAC |
| Fragment 2 for all mutants | tufAall_frag2_rev | GCTGCAGGTCGACCCTTAGCTTAGCCCAGAACTTTAGCAACAACGCCC |
| Fragment 1 for R318A | tufA_R318A_frag1rev | CGGAGTATGCGCGCCGCC |
| Fragment 2 for R318A | tufA_R318A_frag2fwd | GAAGGCGGCGCGCATACT |
| Fragment 1 for R318K | tufA_R318K_frag1rev | AAGAACGGAGTATGTTTGCCGCCTTCATCTTTG |
| Fragment 2 for R318K | tufA_R318K_frag2fwd | CAAAGATGAAGGCGGCAAACATACTCCGTTCTT |
| Fragment 1 for R318N | tufA_R318N_frag1rev | TGAAGAACGGAGTATGGTTGCCGCCTTCATCTT |
| Fragment 2 oforf R318N | tufA_R318N_frag2fwd | AAGATGAAGGCGGCAACCATACTCCGTTCTTCA |
| Fragment 1 of H319A | tufA_H319A_frag1rev | GAACGGAGTCGCACGGCC |
| Fragment 2 for H319A | tufA_H319A_frag2fwd | GGCGGCCGTGCGACTCCG |
| Fragment 1 for E378A | tufA_E378A_frag1rev | ACGGCCGCCCGCACGGAT |
| Fragment 2 for E378A | tufA_E378A_frag2fwd | GCAATCCGTGCGGGCGGC |
| Fragment 1 for R318AH319A | tufA_R318AH319A dbmut_frag1rev | GAACGGAGTCGCCGCGCC |
| Fragment 2 for R318AH319A | tufA_R318AH319A dbmut_frag2fwd | GAAGGCGGCGCGGCGACT |

1 Baba T, Ara T, Hasegawa M, Takai Y, Okumura Y, Baba M, Datsenko KA, Tomita M, Wanner BL, Mori H. 2006. Construction of Escherichia coli K-12 in-frame, single-gene knockout mMutants: tThe Keio collection. Mol Syst Biol 2:2006. <https://doi.org/10.1038/msb4100050>

2 Kitagawa M, Ara T, Arifuzzaman M, Ioka-Nakamichi T, Inamoto E, Toyonaga H, Mori H. 2005. Complete set of ORF clones of Escherichia coli ASKA library (a complete set of E. coli K-12 ORF aArchive): uUnique resources for biological research. DNA Res 12:291–299. https://doi.org/10.1093/dnares/dsi012
